# *Helicobacter pylori* Lipopolysaccharide Is Synthesized via a Novel Pathway with an Evolutionary Connection to Protein *N*-Glycosylation

**DOI:** 10.1371/journal.ppat.1000819

**Published:** 2010-03-19

**Authors:** Isabelle Hug, Marc R. Couturier, Michelle M. Rooker, Diane E. Taylor, Markus Stein, Mario F. Feldman

**Affiliations:** 1 Department of Biological Sciences, University of Alberta, Edmonton, Alberta, Canada; 2 Department of Medical Microbiology and Immunology, University of Alberta, Edmonton, Alberta, Canada; University of Pennsylvania, United States of America

## Abstract

Lipopolysaccharide (LPS) is a major component on the surface of Gram negative bacteria and is composed of lipid A-core and the O antigen polysaccharide. O polysaccharides of the gastric pathogen *Helicobacter pylori* contain Lewis antigens, mimicking glycan structures produced by human cells. The interaction of Lewis antigens with human dendritic cells induces a modulation of the immune response, contributing to the *H. pylori* virulence. The amount and position of Lewis antigens in the LPS varies among *H. pylori* isolates, indicating an adaptation to the host. In contrast to most bacteria, the genes for *H. pylori* O antigen biosynthesis are spread throughout the chromosome, which likely contributed to the fact that the LPS assembly pathway remained uncharacterized. In this study, two enzymes typically involved in LPS biosynthesis were found encoded in the *H. pylori* genome; the initiating glycosyltransferase WecA, and the O antigen ligase WaaL. Fluorescence microscopy and analysis of LPS from *H. pylori* mutants revealed that WecA and WaaL are involved in LPS production. Activity of WecA was additionally demonstrated with complementation experiments in *Escherichia coli*. WaaL ligase activity was shown *in vitro*. Analysis of the *H. pylori* genome failed to detect a flippase typically involved in O antigen synthesis. Instead, we identified a homolog of a flippase involved in protein *N*-glycosylation in other bacteria, although this pathway is not present in *H. pylori*. This flippase named Wzk was essential for O antigen display in *H. pylori* and was able to transport various glycans in *E. coli*. Whereas the O antigen mutants showed normal swimming motility and injection of the toxin CagA into host cells, the uptake of DNA seemed to be affected. We conclude that *H. pylori* uses a novel LPS biosynthetic pathway, evolutionarily connected to bacterial protein *N*-glycosylation.

## Author Summary

Bacterial surfaces are decorated with glycans. The human stomach pathogen *Helicobacter pylori* exposes lipopolysaccharide (LPS) containing Lewis antigens that mimic human glycan structures. *H. pylori* alters its Lewis antigen display in adaptation to the individual host. Lewis antigens can interact with human dendritic cells, thereby inducing a suppression of the immune response and facilitating a chronic *H. pylori* infection. Whereas three general LPS biosynthesis pathways are known, the route of LPS assembly in *H. pylori* remained to be elucidated. We identified and characterized two components of the *H. pylori* LPS pathway, WecA and WaaL, which demonstrated that, as in other bacteria, the glycan is initially assembled onto a polyprenoid lipid carrier. This intermediate then has to cross a membrane barrier, requiring specialized translocases. *H. pylori* does not employ a translocase from common LPS pathways. We show that instead *H. pylori* uses a translocase named Wzk, which is involved in protein *N*-glycosylation in other bacteria. Wzk was able to translocate various glycan structures. The identification of Wzk as the *H. pylori* translocase involved in LPS biosynthesis indicates an evolutionary connection between LPS and glycoprotein biosynthesis pathways.

## Introduction

Lipopolysaccharide (LPS) is a prevalent macromolecule in the outer membrane of Gram negative bacteria and represents an important virulence factor. LPS is composed of three parts: lipid A which is embedded in the outer membrane, the core oligosaccharide, and the O antigen [1]. Lipid A is also known as endotoxin, which refers to the induction of fatal reactions of the human immune system at very low LPS concentrations. Bound to lipid A is the core oligosaccharide, which is relatively well conserved among closely related bacteria. The O antigen represents the outermost region of the LPS.

The O antigen of *Helicobacter pylori* contributes in several respects to the virulence of this human gastric pathogen, which is recognized by the World Health Organization as a Type 1 carcinogen [2]. *H. pylori* mimics carbohydrate structures present on human epithelial cells, blood cells, and in secretions, by incorporating Lewis antigens on its O chains [3]. In most strains, both, Lewis x (Le^x^) and Lewis y (Le^y^), can be found in certain regions of the O antigen. Some strains also display Lewis a (Le^a^) and b (Le^b^) antigens or can have alternative O antigen structures [4]. *H. pylori* profits from this molecular mimicry, as Le^x^ and Le^y^ interact with the C-type lectin DC-SIGN on dendritic cells, which signals the immune system to down-regulate an inflammatory response [5]. The amounts of Lewis antigens and their location on the *H. pylori* O polysaccharide are variable, differing between strains and also between cells from the same isolate [3],[6]. This is due to the phase variable expression of the *H. pylori* fucosyltransferases, enzymes required for the synthesis of Lewis antigens [7]. Evidence suggests that the O antigen structures of *H. pylori* strains are adapted to the individual human host, enabling the establishment of a chronic infection [8],[9].

Unlike most bacteria, the genes involved in LPS biosynthesis in *H. pylori* are not arranged in a single cluster, but rather found in various locations distributed throughout the chromosome. Nevertheless, many enzymes required for *H. pylori* LPS biosynthesis have been identified and characterized. These include glycosyltransferases responsible for the addition of the monosaccharide building blocks in the assembly of the O polysaccharide [4], as well as several proteins involved in the synthesis and modification of the lipid A-core [10]. The pathway used for the assembly and translocation of the O antigen in *H. pylori* remained uncharacterized.

In all characterized LPS biosynthetic pathways, the O polysaccharide is assembled onto the undecaprenyl phosphate (UndP) lipid carrier by specific glycosyltransferases located in the cytoplasmic compartment of the bacteria [1]. Several initiating enzymes have been characterized that transfer a sugar phosphate from a nucleotide activated donor to UndP, forming a pyrophosphate linkage. One of the common initiating enzymes is WecA, a UDP-GlcNAc:undecaprenyl-phosphate GlcNAc-1-phosphate transferase [11],[12]. Other glycosyltransferases sequentially add monosaccharides at the non-reducing end of the growing glycan chain. The lipid-linked glycan is subsequently translocated to the periplasm where the O polysaccharide is transferred from undecaprenyl pyrophosphate (UndPP) onto the lipid A-core. This last step is catalyzed by the O antigen ligase WaaL.

Three LPS biosynthesis pathways are known to date [1]. They are distinguished by three different mechanisms for O antigen polymerization and translocation. In the polymerase-dependent pathway, only short O antigen subunits are assembled in the cytoplasm. These subunits are translocated to the periplasm by the flippase Wzx, where they are polymerized by Wzy with assistance of the chain length regulator Wzz, before the complete O antigen is transferred to the lipid A-core. In the two remaining pathways, the ABC transporter-dependent and the synthase-dependent pathway, the entire O polysaccharide is synthesized at the cytoplasmic side of the inner membrane. The flippase in the ABC transporter-dependent pathway consists of two different polypeptides, Wzm and Wzt. Wzm forms a channel in the inner membrane for the passage of the lipid-linked O antigen, and Wzt provides energy through its ATPase activity. The C-terminal domain of Wzt is required for substrate recognition, and often displays specificity towards the structure of the endogenous O chain [13]. In the third pathway, the key enzyme is the synthase WbbF, which has glycosyltransferase activity and is also required for the translocation of the UndP-linked O antigen to the periplasm [1]. Some exopolysaccharides and capsules are synthesized via pathways that resemble one of these three LPS biosynthesis pathways [14].

In some Gram negative bacteria, including *Campylobacter jejuni* which is closely related to *H. pylori*, the cell surface is covered with lipooligosaccharides (LOS) instead of LPS [1]. LOS and LPS are equivalent macromolecules; however, LOS lacks the O polysaccharide and is limited to a short oligosaccharide bound to the lipid A-core [1]. Generally, the oligosaccharide moiety is directly assembled onto the lipid A in the cytoplasm and UndP is not required for LOS biosynthesis.

The goal of this investigation was to determine the pathway used by *H. pylori* for the assembly of the Lewis antigens onto the lipid A-core. We found that these polysaccharides are assembled as typical O antigens onto the UndP carrier. Surprisingly, for the membrane translocation of the lipid-linked glycan, *H. pylori* employs an enzyme which has not been previously found to be involved in LPS synthesis, but instead is used by other bacteria in the biosynthesis of *N*-glycoproteins. This translocase, named Wzk, has no strict structural requirement for its substrates, a characteristic that enables *H. pylori* to produce O antigens of various structures and lengths according to the phenotype of the infected host.

## Results

### The *H. pylori* genome encodes common LPS biosynthesis enzymes: WecA and WaaL

In order to identify genes possibly involved in *H. pylori* LPS biosynthesis, a genome search was performed using the sequences of enzymes known to participate in LPS biosynthetic pathways. The search resulted in the identification of homologs of the *E. coli wecA* and *waaL* genes. The proteins encoded in the *H. pylori* genes JHP1488 in strain J99 and HPG27_1518 in strain G27 are 22% identical to *E. coli* WecA and 94% identical to each other. The genes JHP0385 and HPG27_389 encode polypeptides which are 19% identical to the O antigen ligase WaaL and 95% identical to each other. The overall homology between WecA and WaaL sequences from different organisms is low. Nevertheless these proteins, including the *H. pylori* homologs identified, share similar membrane topologies and a few conserved key residues. Alignments of the protein sequences are shown in Figure S1 and S2. Intriguingly, the *H. pylori* genome seemed to lack homologs of *wzx*, *wzt*, *wzm* or *wbbF*, which encode the flippase proteins involved in O antigen translocation in the known pathways. Furthermore, sequences encoding an O antigen polymerase Wzy, or a chain length regulator Wzz could not be found. Thus, the canonical LPS pathways are incomplete in *H. pylori*.

To address whether the identified genes were indeed involved in *H. pylori* LPS biosynthesis, mutants in both *H. pylori* strains, J99 and G27, were constructed by inserting a chloramphenicol-resistance cassette into the putative *wecA* and *waaL* open reading frames. For the generation of complemented strains, each gene was re-introduced into the *recA* gene on the chromosome of the corresponding mutant strain. This location in the genome was selected to prevent further recombinations, a procedure expected to stabilize the mutations. We took advantage of the *H. pylori* natural competence for the construction of the mutant strains. Interestingly, this procedure was not successful for the generation of complemented strains, as no colonies were recovered on the selective plates after transformation. However, complemented cells were efficiently obtained by electroporation.

**Figure 1:**
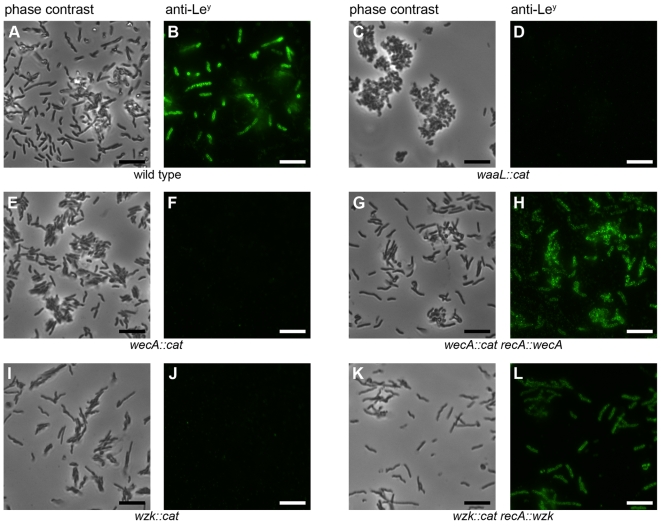
*H. pylori wecA*, *waaL* and *wzk* mutant cells display no Lewis antigens. Fluorescence microscopy of *H. pylori* G27 was performed using monoclonal antibodies against Lewis antigens. Panels A, C, E, G, I and K show phase contrast images, whereas panels B, D, F, H, J and L show bacteria displaying Le^y^ based on reaction with the anti-Le^y^ antibody. Equivalent results were obtained with the anti-Le^x^ antibody (not shown). Lewis antigens are not present on all wild type *H. pylori* cells due to phase variable fucosyltransferases. The bars in the lower right corners of the pictures indicate 10 µm. The images are representative for the results obtained from three independent experiments.

Monoclonal antibodies reacting with Lewis antigens were used to visualize the presence or absence of O antigens on the cell surfaces by fluorescence microscopy (Figure 1). Not all wild type cells reacted with the antibody (Figure 1B). This is due to the high frequency of phase variation in the fucosyltransferase genes (0.2–0.5%) reported by Appelmelk *et al*. [15]. Lewis antigens could also be detected on flagella (Figure S3), confirming the presence of LPS in the membranous sheaths covering these organelles [16]. This was shown previously by electron microscopy [17] but, to our knowledge, not by fluorescence microscopy. Importantly, all the mutant cells were devoid of Lewis antigens (Figure 1D, F), suggesting the participation of the putative WecA and WaaL in *H. pylori* LPS biosynthesis.

**Figure 2:**
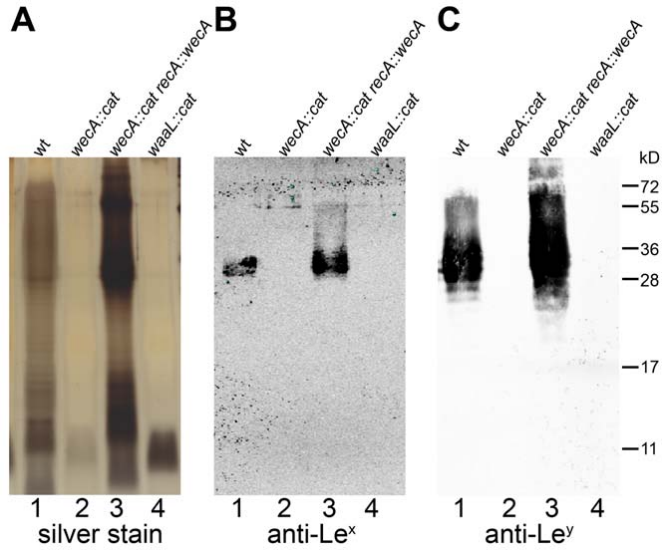
LPS of *H. pylori wecA* and *waaL* mutant strains contains no O antigens. Purified LPS of (1) *H. pylori* G27 wild type, (2) *wecA* mutant, (3) *wecA* complemented and (4) *waaL* mutant strains were analyzed by (A) silver staining, and Western blotting using (B) monoclonal anti-Le^x^ and (C) monoclonal anti-Le^y^ antibodies. Protein marker standards were included for reference. Unlike the smooth LPS of the wild type and complemented strains, mutants expressed lipid A-core without O antigens. Comparable results were obtained with purified LPS from *H. pylori* J99 strains (not shown).

To obtain further evidence for the involvement of the putative WecA and WaaL in *H. pylori* LPS biosynthesis, the LPS of all strains was purified, separated by SDS-PAGE and visualized by silver staining (Figure 2A) and Western blotting, using monoclonal anti-Le^x^ (Figure 2B) and anti-Le^y^ antibodies (Figure 2C). Only rough LPS without O chains and Lewis antigens was produced by the mutant strains (Figure 2, lanes 2, 4), demonstrating that both targeted genes are essential for O antigen display. However, whereas the complementation of the putative *wecA* mutants was successful, the production of smooth LPS was restored only at minimal levels after reintroduction of the putative *waaL* gene into the chromosome of the *waaL* mutant strain (Figure S4).

Based on the evidence presented we annotated JHP1488 and HPG27_1518 as *wecA_HP_*. Similarly, JHP0385, as well as HPG27_389 were named *waaL_HP_*.

### WecA_HP_ is active in *E. coli*

**Figure 3:**
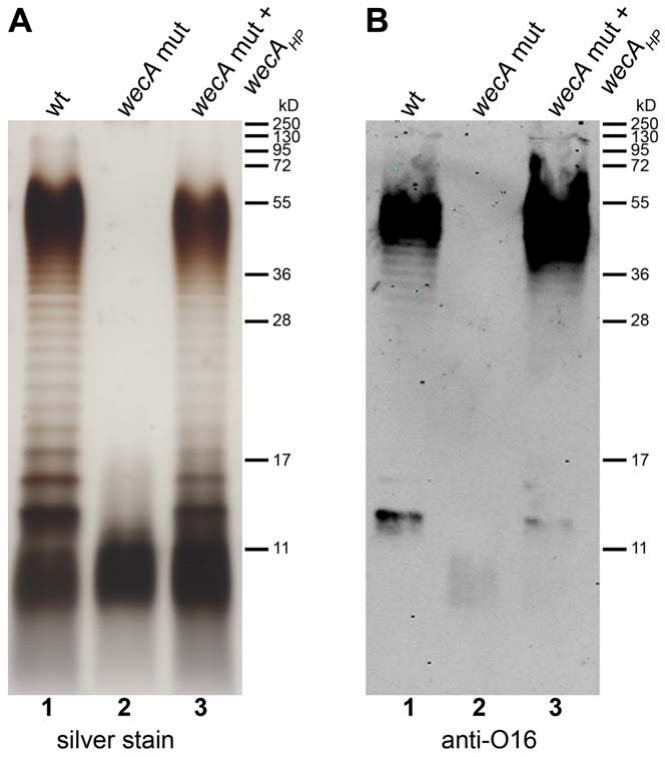
Demonstration of WecA_HP_ activity in *E. coli*. Purified LPS from (1) *E. coli* parental strain W3110 transformed with pMF19, encoding a functional rhamnosyltransferase, (2) *E. coli wecA* mutant strain CLM37 transformed with pMF19 and empty vector pEXT20 and (3) CLM37 transformed with pMF19 and pIH22 containing *wecA_HP_*, were analyzed by (A) silver staining and (B) Western blotting using an antibody recognizing the *E. coli* O16 antigen. Protein marker standards were included for reference.

The availability of *E. coli* strains with specific mutations in LPS biosynthesis genes allowed us to test for activity of WecA_HP_ and WaaL_HP_ by recombinant expression in these mutant strains. *E. coli* strains carrying either a mutation in *wecA* or *waaL* were transformed with plasmids carrying the corresponding *H. pylori* homolog gene (pIH22 and pIH52, respectively). As shown in Figure 3, *wecA_HP_* complemented O antigen synthesis in the *E. coli wecA* mutant, which confirms its role as a UDP-GlcNAc: undecaprenyl-phosphate GlcNAc-1-phosphate transferase.

On the contrary, although WaaL_HP_ could be expressed in *E. coli*, it was unable to restore smooth LPS production in an *E. coli waaL* mutant (data not shown). The transfer of O antigens onto the lipid A-core generally requires a strain specific core structure, and heterologous expression of O antigen ligases is therefore often not functional [1],[18],[19]. *E. coli* and *H. pylori* core regions are structurally different [20], which could explain the lack of activity of WaaL_HP_ in *E. coli*.

### *H. pylori* WaaL is active *in vitro*

**Figure 4:**
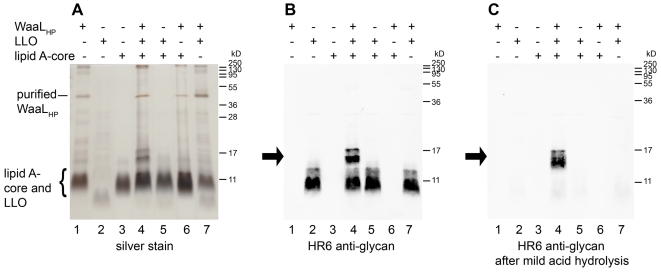
Demonstration of WaaL_HP_ activity *in vitro*. Mixtures with purified WaaL_HP_, *H. pylori* lipid A-core (acceptor) and *C. jejuni* UndPP-linked heptasaccharide (LLO; substrate) were incubated overnight. (1) WaaL_HP_ only, (2) substrate only, (3) acceptor only, (4) all components, (5) no WaaL_HP_, (6) no substrate, (7) no acceptor. Reaction products were detected by (A) silver staining and (B) Western blotting using an antibody reactive against the *C. jejuni* heptasaccharide (HR6). (C) Western blotting after removal of the substrate by mild acid hydrolysis confirmed the presence of heptasaccharide attached to lipid A-core, as this reaction product is not affected by the acid treatment. Arrows indicate the reaction product. Contaminating *E. coli* lipid A-core co-purified with WaaL_HP_ (A, lane 1, 4, 6, 7). Note that the *E. coli* and *H. pylori* lipid A-cores as well as the *C. jejuni* LLO present similar electrophoretic mobilities. Protein marker standards were included for reference.

As full complementation of the *H. pylori waaL_HP_* mutant strain could not be achieved and the enzyme was not functional with the *E. coli* lipid A-core as an acceptor, we tested O antigen ligation activity by performing an *in vitro* assay. WaaL_HP_ containing a C-terminal deca-His tag (encoded in pIH52) was expressed in the *E. coli waaL* mutant strain CLM24 [21]. Expression in this strain was selected to prevent possible contamination with the *E. coli* O antigen ligase. Membranes containing the enzyme were solubilized with detergents and WaaL_HP_ was purified by nickel affinity chromatography as described in Material and Methods (Figure S5). As the band corresponding to purified protein had an apparent molecular weight of about 36 kD instead of the expected 50 kD, the identity of the polypeptide was confirmed by mass spectrometry. As seen with silver staining (Figure 4A, lane 1), lipid A-core from *E. coli* co-purified with the *H. pylori* ligase. Lipid A-core from the *H. pylori waaL_HP_* mutant strain was purified and used as an acceptor structure. O antigen ligases typically show relaxed specificity towards the structure of the O polysaccharide [1], a property that allows the use of diverse UndPP-linked glycans as substrates for the *in vitro* assay. Due to the presence of multiple bands, LPS containing a polymerized O antigen is often indistinguishable from the corresponding UndPP-bound O antigen in SDS-PAGE and Western blot analysis. We reasoned that the use of a short oligosaccharide of defined length would facilitate the interpretation of results. Therefore, the UndPP-linked heptasaccharide derived from the *C. jejuni N*-linked protein glycosylation pathway was selected as substrate. This lipid-linked glycan can be synthesized in *E. coli* cells carrying the plasmid pACYC*pglBmut* [22], which contains all the enzymes required for the assembly of the heptasaccharide, and has been successfully shown to be a suitable substrate for *in vitro* glycosylation [23],[24].

All reactants were mixed and incubated at 37°C overnight. Reaction products appeared as bands of higher molecular weight in SDS-PAGE (Figure 4A, lane 4) and positively reacted with the HR6 antibody, which recognizes the *C. jejuni* heptasaccharide (Figure 4B, lane 4). The reaction mixtures were subjected to mild acid hydrolysis. In such conditions, the reaction product is stable, whereas the substrate is hydrolyzed (Figure S6). After acid hydrolysis, the HR6-reacting bands corresponding to LPS were still present (Figure 4C, lane 4), whereas the bands corresponding to the UndPP-linked heptasaccharide substrate were no longer observed (Figure 4C, lanes 2, 4, 5, 7). These results demonstrated the successful transfer of the *C. jejuni* heptasaccharide onto the *H. pylori* lipid A-core acceptor, thereby confirming the O antigen ligase activity of WaaL_HP_. The *E. coli* lipid A-core present in the fraction containing the recombinant *H. pylori* ligase was not an appropriate WaaL_HP_ acceptor, as activity was dependent on the presence of *H. pylori* lipid A-core (Figure 4, lanes 4, 7).

It is notable that unlike the O antigen ligase of *Pseudomonas aeruginosa* [19], WaaL_HP_ did not require ATP as an energy source. The presence or absence of ATP in the *in vitro* reaction did not noticeably affect ligation efficiency (Figure S7).

### *H. pylori* encodes an O antigen translocase usually involved in protein *N*-glycosylation

From the previous experiments we concluded that the *H. pylori* Lewis antigens are synthesized onto the UndPP carrier via a pathway initiated by WecA_HP_ and involving WaaL_HP_ for the transfer of the glycans onto the lipid A-core. It was puzzling that no gene encoding one of the common O antigen translocases could be found in the *H. pylori* genome. Therefore, we searched for the presence of alternative flippases belonging to other biosynthetic pathways.

A gene homolog to *pglK* (formerly named *wlaB*) which encodes a flippase involved in the translocation of the UndPP-linked heptasaccharide during protein *N*-glycosylation in *C. jejuni* [25] was found in the *H. pylori* genome. The genes annotated as JHP1129 in strain J99 and HPG27_1153 in strain G27 encode polypeptide sequences which are 97% identical to each other and share 37% identity with *C. jejuni* PglK (see alignments in Figure S8). No evidence for the presence of *N*-glycoproteins has been found in *H. pylori*, and therefore, the homolog of *C. jejuni* PglK was our principal candidate as the *H. pylori* O antigen translocase.

**Figure 5:**
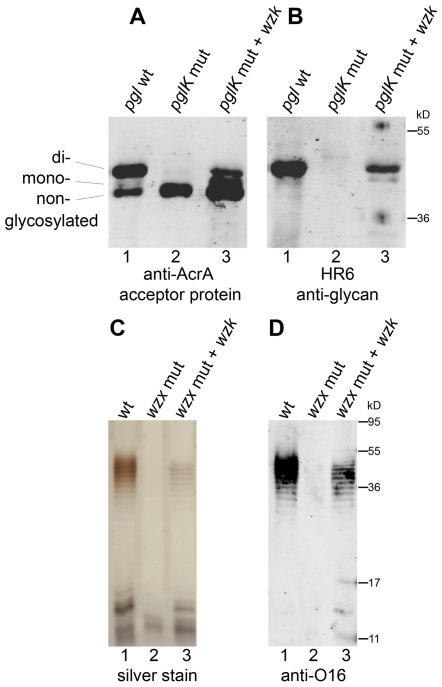
*H. pylori* has a flippase able to translocate diverse UndPP-linked glycans. Activity of the *H. pylori* translocase was examined with complementation assays in flippase deficient *E. coli*. (A, B) Glycosylation of the acceptor protein AcrA in the *N*-glycosylation pathway of *C. jejuni* was analyzed by Western blotting with (A) anti-AcrA antibody and (B) an antibody (HR6) recognizing the *C. jejuni* glycan. *C. jejuni pglK* was (1) functional, (2) mutated, or (3) mutated and complemented by *H. pylori wzk*. (C, D) Purified LPS of *E. coli* strains containing (1) a functional flippase Wzx, (2) a *wzx* mutation or (3) a *wzx* mutation complemented by *wzk*, was visualized by silver staining and Western blotting using an antibody recognizing the *E. coli* O16 antigen. Protein marker standards were included for reference.

We investigated the flippase activity of the putative *H. pylori* translocase through complementation experiments in *E. coli* described by Alaimo *et al*. [25]. A gene cluster encoding the complete *C. jejuni N*-linked protein glycosylation machinery, which includes the PglK flippase, was introduced into an *E. coli* mutant strain devoid of known glycan flippases. These cells were also transformed with plasmid pIH18 expressing AcrA, a *C. jejuni* acceptor protein that carries two *N*-glycosylation sites. Glycosylation of AcrA in *E. coli* was detected through the appearance of two extra bands of higher molecular weight in Western blots, immunoreactive to the AcrA and the *C. jejuni* glycan-specific HR6 antibodies (Figure 5A, B). Glycosylation of AcrA was abolished when the translocase PglK was absent (Figure 5A, B, lane 2), but was restored in the presence of the putative *H. pylori* translocase encoded in pIH23 (Figure 5A, B, lane 3), indicating that the activities of PglK and its *H. pylori* homolog (named Wzk, according to the current official nomenclature of LPS genes [26]) are interchangeable. Further evidence of the Wzk translocase activity was obtained by demonstrating its capability to restore the flipping of O antigen in an *E. coli wzx* mutant strain (Figure 5C, D, lane 3). Taken together, these results indicate that Wzk is a translocase for UndPP-linked glycans, equivalent to PglK. Structurally different glycans were translocated by Wzk (Figure S9), indicating a relaxed substrate specificity of this enzyme.

**Figure 6:**
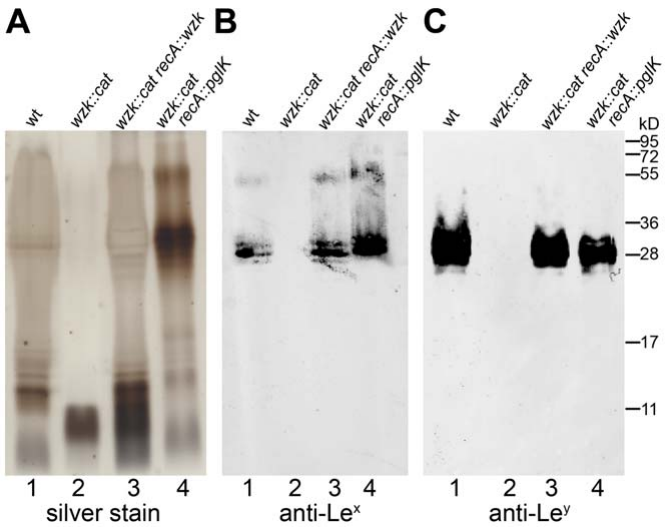
Wzk is the *H. pylori* O antigen flippase and can be replaced by *C. jejuni* PglK. Purified *H. pylori* LPS was analyzed by (A) silver staining and Western blotting using (B) anti-Le^x^ and (C) anti-Le^y^ monoclonal antibodies. Lane 1 shows smooth LPS extracted from wild type *H. pylori* G27 cells; lane 2 LPS from the G27 *wzk* mutant strain not containing O antigens; smooth LPS production is restored in the *wzk* complemented strain shown in lane 3. Shown in lane 4: the *C. jejuni* flippase PglK can functionally complement the *H. pylori* Wzk, and restore a smooth LPS phenotype in the *H. pylori* G27 *wzk* mutant. Protein marker standards were included for reference.

To demonstrate the involvement of Wzk in *H. pylori* LPS biosynthesis, the *wzk* gene was mutated as described previously for *wecA_HP_* and *waaL_HP_*. Fluorescence microscopy showed that Lewis antigens were not present on the surface of *H. pylori wzk* mutant cells (Figure 1J). The absence of Le^x^ and Le^y^ antigens on purified LPS from the mutant strain was shown by Western blot analysis (Figure 6B, C, lane 2). SDS-PAGE followed by silver staining showed that the LPS of the *wzk* mutant did not contain O antigens (Figure 6A, lane 2). Upon complementation with either *wzk* or its homolog *pglK*, the synthesis of smooth LPS was restored (Figure 6, lanes 3, 4). Taken together, these results demonstrated that Wzk is an essential component in the LPS biosynthesis pathway in *H. pylori*, responsible for the translocation of the O antigen. Although in their native hosts PglK and Wzk participate in different pathways, both enzymes can be functionally exchanged, being able to translocate glycans of diverse structures and lengths.

### O antigen is not required for *H. pylori* motility

Our fluorescence microscopy experiments allowed us to confirm the presence of LPS on the membranous sheaths covering *H. pylori* flagella, raising the possibility of a connection between LPS integrity and motility. We tested this hypothesis by comparing the swimming activity of the O antigen deficient strains relative to the wild type strains. After growth on soft agar plates, no significant difference between the diameters of colony expansion was detected (results not shown). It was concluded that the absence of O antigens in the *H. pylori* LPS has no adverse effect on flagellar function *in vitro*.

### *H. pylori* O antigen mutation may adversely affect selected type IV secretion systems

As mentioned above, the protocol for natural DNA uptake was not successful in the construction of the complemented strains. One possible reason is a reduced natural competence of the *H. pylori* O antigen mutants. The natural competence of *H. pylori* depends on the ComB type IV secretion system [27]. As *H. pylori* possesses additional type IV secretion systems, we examined if the presence of O antigens on the bacterial surface might be required for the function of these machineries. One additional *H. pylori* type IV secretion system is encoded in the *cag* pathogenicity island [28]. The presence of this gene cluster in *H. pylori* correlates with enhanced virulence, as the Cag type IV secretion system builds a needle-like device for the injection of a single known effector protein, CagA, directly into the host cells. Within the host, CagA is tyrosine phosphorylated and interferes with cell signaling pathways [28].

**Figure 7:**
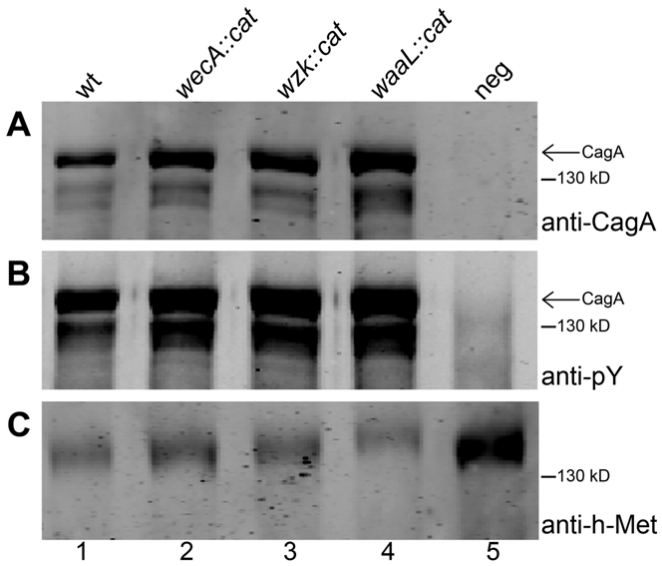
CagA translocation is functional in O antigen negative *H. pylori* strains. Human gastric epithelial AGS cells were infected with *H. pylori* G27 (1) wild type, (2) *wecA* mutant, (3) *wzk* mutant, or (4) *waaL* mutant strains. (5) Non-infected AGS cells served as negative control. AGS membranes were examined for translocation and tyrosine phosphorylation of CagA by Western blotting using (A) anti-CagA antibody and (B) anti-phosphotyrosine (pY) antibody. (C) The host membrane marker h-Met was detected with an anti-h-Met antibody for a loading control. Arrows indicate full length phosphorylated CagA. A protein marker standard was included for reference.

We compared the efficiency of CagA injection into human gastric epithelial cells between wild type and O antigen mutant *H. pylori* strains. AGS gastric epithelial cells were infected with *H. pylori* cells from an overnight grown liquid culture. Cells were harvested four hours after infection, when many epithelial cells displayed an elongated morphology, a typical effect following CagA translocation [28]. AGS cell membrane fractions were collected and analyzed by Western blotting, using anti-CagA (Figure 7A) and anti-phosphotyrosine (Figure 7B) antibodies. All *H. pylori* strains injected similar amounts of CagA. We concluded that mutations interfering with the synthesis of smooth LPS in *H. pylori* may reduce natural competence, but do not affect type IV secretion systems in general.

## Discussion

**Figure 8:**
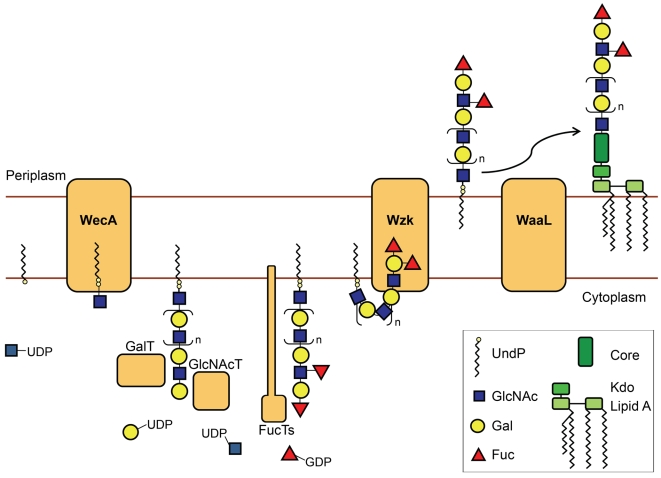
Novel LPS biosynthesis pathway in *H. pylori*. The figure shows a simplified illustration of the *H. pylori* LPS biosynthesis pathway. The *H. pylori* O chains are assembled in the cytoplasm onto a polyisoprenoid membrane anchor. The O antigen synthesis is initiated by the UDP-GlcNAc: undecaprenyl-phosphate GlcNAc-1-phosphate transferase WecA. Processive glycosyltransferases alternately add Gal and GlcNAc residues, producing the linear O chain backbone. Fucosyltransferases then attach Fucose residues on selected locations of the O antigen backbone, generating Lewis antigens. The flippase Wzk transfers the O polysaccharide to the periplasm, where it is attached onto the lipid A-core by the action of the O antigen ligase WaaL. The LPS molecule can then be transported to the outer leaflet of the *H. pylori* outer membrane.

With the display of Lewis antigens on the O chains, the LPS plays a unique role in *H. pylori* colonization. The fact that the genes involved in LPS biosynthesis are not grouped in a single locus in the *H. pylori* chromosome is probably one of the reasons why the pathway for the biosynthesis of this key macromolecule remained to be determined. The objective of this investigation was to elucidate the LPS biosynthetic pathway in *H. pylori*. Genomic analysis revealed that none of the previously characterized pathways for LPS biosynthesis is complete in *H. pylori*, suggesting that this bacterium uses an alternative strategy. One possibility was that the synthesis of LPS took place directly on the lipid A-core as it occurs with the LOS biosynthesis in *C. jejuni* and *Neisseria* spp. [1]. However, using a combination of genetic, biochemical and microscopy techniques, we showed that the O antigen is assembled onto a polyisoprenoid lipid carrier. Figure 8 illustrates our model of *H. pylori* LPS biosynthesis. WecA_HP_ initiates this pathway by transferring a GlcNAc-phosphate from UDP-GlcNAc to UndP. The resulting molecule, UndPP-GlcNAc, serves as an acceptor for the assembly of the O chain backbone, composed of alternating GlcNAc and Gal residues. Some of these linear polysaccharides are decorated at selected locations through the activity of various fucosyltransferases, producing the Lewis antigens [4]. After translocation to the periplasm by Wzk, the O chain is attached onto the lipid A-core acceptor by the action of the O antigen ligase WaaL_HP_.

*H. pylori* LPS biosynthesis follows a novel pathway, differing from all the established LPS pathways in the translocation of the O chain. We found that this step is accomplished by Wzk, which is not related to any described translocase involved in O antigen synthesis. Instead, Wzk is homolog to *C. jejuni* PglK, responsible for UndPP-heptasaccharide flipping during protein *N*-glycosylation. Wzk and PglK are related to the lipid A-core flippase MsbA, with the closest sequence similarity among known ATP transporters. ATPase activity of PglK has been reported by Alaimo *et al.* [25]. As the C-terminal Walker domains are well conserved between PglK and Wzk, Wzk most likely also possesses ATPase activity. All ABC transporter-dependent LPS pathways described to date require two polypeptides, Wzm and Wzt, for the translocation of the O chains [1]. The only homology of these proteins to Wzk is found in the ATP binding domains of Wzt. Wzm possesses several transmembrane domains and is proposed to form a channel in the inner membrane. Wzt provides the energy for the flipping mechanism through its ATPase activity. In *H. pylori*, the flippase Wzk is the only polypeptide required and sufficient for translocation of UndPP-linked glycans. The ability of Wzk to translocate Lewis antigens, the *C. jejuni* heptasaccharide, as well as the *E. coli* O16 antigen, demonstrates that Wzk activity is independent of the length or the composition of the translocated UndPP-linked sugars.

In most bacteria, the genes involved in O antigen biosynthesis are clustered in a single locus, which facilitates horizontal gene exchange and regulation of O antigen synthesis [1]. In contrast, the three genes investigated in this work, as well as the other genes involved in O antigen biosynthesis in *H. pylori*, are located in separate loci dispersed along the chromosome. *H. pylori* exhibits a high rate of DNA uptake and genetic variability [29]. An independent location of the genes requires individual gene regulation, which could be beneficial for *H. pylori* by allowing more diversity and flexibility in the LPS structure. It is particularly intriguing that the position of the *H. pylori wzk* gene is located in close proximity to *tRNA* genes, which are known to be hot-spots for the insertion of mobile genetic elements [30]. On the contrary in *C. jejuni*, PglK is encoded as part of the *pgl*-cluster, responsible for *N*-linked protein glycosylation [22]. Interestingly, the oligosaccharyltransferase PglB, also encoded in the *pgl*-locus, has homologs in eukaryotes and archaea but not in *H. pylori*, which does not possess this general glycosylation machinery [31]. Different scenarios can be advanced to describe the origin of the Wzk-like translocases. *H. pylori* may have discarded its *pgl*-cluster, yet retained *wzk* to act on the synthesis of LPS. Alternatively, the *wzk* gene could have been adopted by other epsilon- or delta proteobacteria to produce *N*-glycoproteins. Subsequently, some of these organisms, like *C. jejuni*, may have lost their LPS cluster, producing LOS instead. In either case, bacteria appear to favor the dedication of their lipid-linked glycans exclusively to one biosynthetic pathway, either protein glycosylation or LPS biosynthesis.

We encountered difficulties with natural transformation of O antigen mutant *H. pylori* cells and electroporation was required for the construction of strains for complementation experiments. One possible explanation is a loss of natural competence for DNA uptake. A similar observation has been reported for a *C. jejuni* LOS mutant strain [32]. However, in another study *C. jejuni* LOS and capsule mutants displayed increased DNA uptake ability [33]. Although the exact role of O chains in *H. pylori* natural competence remains unclear, we demonstrated that the presence of O antigens does not have a general inhibitory effect on *H. pylori* type IV secretion systems, because the type IV secretion apparatus encoded in the *cag* pathogenicity island was functional in O chain deficient *H. pylori* mutants. Alternatively, the lack of O antigens may induce a stress response, resulting in the induction of DNA restriction enzymes, which could digest the foreign DNA before or after uptake into the cells.

Due to the presence of LPS on the *H. pylori* flagella, we investigated the possible role of O antigens in motility. *H. pylori* mutant strains were not defective in *in vitro* swimming motility compared to wild type strains. However, the presence of LPS on the flagellar surface may still play a role *in vivo*. The O antigens may have a protective function by shielding the flagella against components of the host immune defense, and by actively down-regulating flagellin-specific activation of the innate immune system via the interaction between Lewis antigens and DC-SIGN.

A central role of the Lewis antigens in *H. pylori* pathogenicity is their interaction with DC-SIGN, which results in modulation of the host immune defense [5]. As Lewis antigen expression is phase variable due to the reversible switching off of the fucosyltransferases, *H. pylori* maintains a balance between activation and repression of the host immune system [9]. In addition, the fucosylated locations along the O chain backbone are finely adapted to the host phenotype [8]. With this mechanism, a permanent infection can be established, which in rare cases results in gastric cancer. The O antigen translocase Wzk, which we show here is essential for the cell surface expression of Lewis antigens, could be an attractive target for the design of antibiotics effective against *H. pylori* and possibly *C. jejuni* infections.

Interestingly, Wzk is the first protein common to both, LPS biosynthesis and protein *N*-glycosylation, supporting an evolutionary connection between these pathways.

## Materials and Methods

### Genome analysis

NCBI BLASTP (default settings) was used for the search of putative WecA, WaaL and lipid-linked glycan translocase polypeptide sequences encoded in the *H. pylori* genome. Global sequence identities were calculated with LALIGN (default settings, global) (http://www.ch.embnet.org/software/LALIGN_form.html).

### PCR and plasmid construction

Oligonucleotides used for DNA amplification are listed in Table S1.

#### Plasmids containing *wecA_HP_*

For the construction of pIH22, *wecA_HP_* was amplified by PCR, using the primers WecAHPEcoRIfw and WecAHPH6XbaIrv and genomic DNA from *H. pylori* J99 as template. The *wecA_HP_* PCR product was cloned into pEXT20 [34], using the restriction enzymes EcoRI and XbaI (all restriction enzymes were purchased from New England Biolabs unless indicated otherwise). pIH22 was used for the expression of WecA_HP_ in *E. coli*.

The primers WecA_forward and WecA_reverse were used to amplify *wecA_HP_* from *H. pylori* J99, which was cloned into pGEM-T (Promega), generating pGEM-HPwecA. The obtained plasmid was digested with SgfI (Promega), which is cutting the open reading frame of *wecA_HP_*. Blunt ends were generated with a Klenow fragment (New England Biolabs). Insertion of a chloramphenicol-resistance cassette (CAT), derived from plasmid DT3072 [35] after digestion with HincII, resulted in pGEM-HPwecA-CAT, which was used in the construction of *H. pylori wecA::cat*.

The J99 *wecA_HP_* gene was amplified with the primers NdeIHPJ99wecAfw and HPJ99wecAH6BamHIrev. The PCR product was ligated into vector pGEM-T easy (Promega), resulting in plasmid pIH27, which was subsequently digested with NdeI and BamHI and cloned into vector pSK+recxorf8-flag [36] to obtain pIH42. pIH42 was used in the construction of *H. pylori wecA::cat recA::wecA*.

#### Plasmids containing *waaL_HP_*

Genomic DNA from *H. pylori* J99 was used as template for the PCR amplification of *waaL_HP_* using Ligase_forward and Ligase_reverse primers. The amplified DNA sequence was cloned into pGEM-T, generating pGEM-HPwaaL. The *waaL_HP_* gene was subsequently cut with NheI (Invitrogen) and blunt ends were generated using a polymerase Klenow fragment. The CAT cassette was excised from DT3072 using HincII restriction enzyme and inserted into the *waaL_HP_* gene of pGEM-HPwaaL, generating pGEM-HPwaaL-CAT, which was used for the construction of *H. pylori waaL::cat*.

For the construction of pIH53, *waaL_HP_* from *H. pylori* G27 was amplified with primers NdeIG27waaLfw and G27waaLH10BamHIrv. The PCR product and pSK+recxorf8-flag were digested with NdeI and BamHI and ligated. The resulting plasmid pIH53 was used for the construction of *H. pylori waaL::cat recA::waaL*.

Plasmid pIH53 was digested with NdeI, whereas pEXT20 was digested with EcoRI. Blunt ends were generated with a Klenow fragment and the resulting DNA fragments were further digested with BamHI. Subsequent ligation of the DNA fragments resulted in pIH52, which was used for the expression of WaaL_HP_ in *E. coli*.

#### Plasmids containing *wzk*

The gene encoding the flippase Wzk was amplified from genomic *H. pylori* J99 DNA using the primers KpnIHPpglKfw and HPpglKH6XbaIrev. The PCR product and pEXT20 were digested with KpnI and XbaI. Ligation of the DNA fragments resulted in pIH23, which was used for the expression of Wzk in *E. coli*.

The gene *wzk* in pIH23 was cut with PsiI, and the CAT cassette from HincII-digested DT3072 was inserted by ligation. The resulting plasmid pIH40 was used for the construction of *H. pylori wzk::cat*.

The *wzk* gene was excised from pIH23 through digestion with KpnI and PstI restriction enzymes. Plasmid pSK+recxorf8-flag was digested with BamHI and NdeI. After treatment with a Klenow fragment the restriction products were ligated to generate pIH43, which was used for the construction of *H. pylori wzk::cat recA::wzk*.

#### Plasmids containing *C. jejuni* genes

Oligonucleotides NdeICj81116pglKfw and Cj81116pglKXbaIrv were used for the PCR amplification of *C. jejuni pglK*, with the plasmid pACYC*pgl* [22] serving as template. The PCR product was digested with NdeI and XbaI and ligated into the vector pSK+recxorf8-flag, treated with the same restriction enzymes, leading to plasmid pIH54, used for the construction of *H. pylori wzk::cat recA::pglK*.

The gene *acrA* encoding the glycosylation acceptor protein AcrA from *C. jejuni* was excised from plasmid pWA2 [21] with SfoI and ZraI for ligation into vector pEXT21 [34], which was digested with SmaI. The resulting plasmid was named pIH18, and used for the expression of AcrA in *E. coli*.

### Bacterial strains and growth conditions

*H. pylori* strains J99 [37] and G27 [38] served as parental strains for the construction of O antigen mutants. *H. pylori* mutant strains were generated through natural transformation with the plasmids pGEM-HPwecA-CAT for *wecA* mutants, pGEM-HPwaaL-CAT for *waaL* mutants and pIH40 for *wzk* mutants, respectively, resulting in the disruption of the targeted genes through insertion of a chloramphenicol resistance cassette by homologous recombination. Mutant strains were recovered as single colonies after growth on selective plates containing chloramphenicol. Complementation was achieved through electroporation of the mutant strains for the uptake of the plasmids pIH42 for *wecA* complementation, pIH53 for *waaL* complementation and pIH43 for *wzk* complementation, respectively. Following homologous recombination, the complemented genes were inserted into the chromosome of the *H. pylori* mutant strains, disrupting the *recA* gene. Complemented colonies were selected on plates containing chloramphenicol and kanamycin. All strains were verified with PCR analysis.

*H. pylori* strains were grown on brucella broth agar plates, supplemented with 10% heat inactivated fetal bovine serum, or on brain heart infusion agar with 10% horse serum. The antibiotics vancomycin (5 µg/ml), cycloheximide (100 µg/ml), trimethoprim (10 µg/ml) and amphotericin B (8 µg/ml) were added and cells were incubated at 37°C under micro-aerobic conditions, obtained by adding a CampyGen gas pack (Oxoid) to an anaerobic jar. O antigen mutant strains were selected with chloramphenicol (25 µg/ml). Kanamycin (20 µg/ml) was added for the selection of complemented strains. Liquid cultures were grown overnight in brucella broth supplemented with 10% heat inactivated fetal bovine serum and the appropriate antibiotics at 37°C with 160 rpm rotation. *E. coli* strains were grown overnight in LB broth at 37°C with rotation at 200 rpm.

### Fluorescence microscopy

Microscope cover glasses were prepared for the attachment of cells using (3-aminopropyl)triethoxysilane (Sigma) according to Strähle and coworkers [39]. Overnight *H. pylori* cultures were adjusted to an optical density at 600 nm wave length (OD_600_) of 1.0 per ml. Cells were washed with PBS and an equivalent of 0.4 OD_600_ was applied to each cover glass. After 30 min of incubation on ice, the cell suspension was removed and cells were fixed with 4% paraformaldehyde for 10 min at room temperature. Staining and microscopy procedures were conducted as described by Couturier and Stein [40], whereby a monoclonal anti-Le^y^ antibody (1/200) (Calbiochem) and a secondary Alexa Fluor 488 goat anti-mouse antibody (1/500) (Molecular Probes) were used for staining.

### LPS analysis (immunoblotting/silver staining)

Small scale LPS extraction using hot phenol was performed following the procedure described by Marolda *et al.* [41], with the exception that ethyl ether was replaced by ethanol for the washing of the LPS pellet. The LPS was run on a 15% SDS-PAGE and visualized by the silver staining method described by Tsai and Frasch [42], or by Western blotting, using monoclonal mouse anti-Le^x^ and anti-Le^y^ antibodies (Calbiochem), or rabbit anti-O16 antigen antiserum (Statens Institute, Denmark). After incubation with a secondary goat anti-mouse IgM IRDye-800 antibody or a goat anti-rabbit IRDye-800 antibody, respectively (LI-COR Biosciences), the blots were scanned with an Odyssey infrared imaging system (LI-COR Biosciences).

### Activity tests in *E. coli*

*E. coli* serogroup O16 laboratory strains produce rough LPS without O antigen due to a mutation inactivating the rhamnosyltransferase responsible for the addition of the second sugar residue in the O chain assembly. Smooth LPS production can be restored with the addition of a plasmid pMF19 containing the rhamnosyltransferase gene [43]. *E. coli* W3110 transformed with pMF19 served as a positive control, producing long chain O16 LPS. *E. coli* strain CLM37 has a mutation in *wecA_EC_*, and therefore is unable to assemble O chains [44]. CLM37 was transformed with pMF19 and empty vector pEXT20 to serve as negative control in the experiment testing for WecA_HP_ activity. WecA_HP_ activity was examined in CLM37 transformed with pMF19 and pIH22.

The *E. coli* O antigen ligase mutant strain CLM24 was transformed with pMF19 and either pIH52 or pEXT20 for the analysis of WaaL_HP_ activity or for the corresponding negative control, respectively.

To test the ability of *H. pylori* Wzk to flip UndPP-linked glycans in an *N*-glycosylation pathway, *E. coli* strain SCM7 [25] containing mutations in oligosaccharide translocases was transformed with pIH23 (containing *wzk*), pIH18 (encoding the acceptor protein AcrA) and pACYC*pglKmut* (encoding the *C. jejuni* glycosylation machinery with a mutation in the translocase gene *pglK* [44]). In the negative control strain the empty vector pEXT20 was transformed instead of pIH23. The positive control strain was transformed with pACYC*pgl* (containing the intact *C. jejuni* glycosylation machinery) instead of pACYC*pglKmut*. To further examine if Wzk has O antigen translocase activity, the *E. coli* flippase mutant strain CLM17 [45] was transformed with pMF19 and pIH23, or the vector control pEXT20.

LPS profiles were analyzed by silver staining as described above. Western blotting was used to determine the glycosylation status in the Wzk activity tests. As primary antibodies, either an anti-AcrA antibody [22], recognizing the acceptor protein, or the antiserum HR6 (S. Amber and M. Aebi, manuscript in preparation), reacting with the *C. jejuni* glycan, were applied. After incubation with a secondary goat anti-rabbit IRDye680 antibody (LI-COR Biosciences), bands were visualized with an Odyssey imaging system (LI-COR Biosciences).

### Mass spectrometry

The protein band corresponding to the putative WaaL_HP_ was excised from a coomassie stained gel (Figure S5). The protein was in-gel digested using trypsin (Promega) according to Shevchenko *et al.* [46]. Peptide fragments were eluted from the gel piece, desalted using zip-tip_C18_ (Millipore) according to the supplier protocol and dissolved in 0.1% formic acid. Peptides were separated with a LC/MSD Trap XCT (Agilent Technologies) and the resulting mass spectrum was used for the identification of the protein by the Mascot search engine (www.matrixscience.com) using the NCBInr database.

### WaaL *in vitro* assay

The *E. coli* O antigen ligase mutant CLM24 was transformed with pIH52 which encodes *waaL_HP_* with a C-terminal deca-histidine tag. Cells were grown overnight at 37°C with 0.2 mM IPTG for the production of the O antigen ligase. The protocol described by Faridmoayer *et al.* [24] for the purification of an oligosaccharyltransferase was used for the purification of WaaL_HP_. Briefly, membrane fractions were solubilized with 2% elugent (Calbiochem) in phosphate buffer, pH 7.2. Elugent concentration was diluted to 1% and the membrane fraction loaded unto a nickel agarose column (Qiagen) with 20 mM imidazole. The washing solution contained 50 mM imidazole and 0.5% DDM (Anatrace). Ligase was eluted with 250 mM imidazole in the presence of 0.5% DDM. The lipid A-core from *waaL_HP_* mutant cells was obtained as described above and utilized as acceptor in the *in vitro* assay. UndPP-linked glycans serving as substrates were produced by *E. coli* CLM24 cells transformed with pACYC*pglBmut* [22]. A crude UndPP-glycan extraction was performed according to Ielpi *et al*. [47]. Purified WaaL_HP_ (4–5 µg), purified LPS (1.2 µg, estimated using the method described by Osborn [48]), and an extract containing the UndPP-glycan (20% v/v) were incubated overnight at 37°. The volume was adjusted to 50 µl with reaction buffer as previously described for *in vitro* glycosylation [24] (50 mM Tris-HCl pH 7.5, 100 mM sucrose and 1 mM MnCl_2_), with or without ATP (2 mM). Reaction products were visualized by silver staining or Western blotting using the glycan specific antibody HR6. In addition, mild acid hydrolysis was performed similar to the method described by Ielpi *et al.* [47], by incubation in 1% acetic acid at 80°C for 30 min to destroy the pyrophosphate linkage of the substrate.

### Motility assay

*H. pylori* wild type and O antigen mutant cells from overnight cultures, grown either on plates or in liquid media, were spotted on soft brucella broth agar plates (0.3% agar) and incubated at 37°C under micro-aerobic conditions. Swimming motility was analyzed after 3–6 days by comparison of the colony growth diameters.

### CagA translocation assay

The procedure for the CagA translocation assay was previously described by Cendron *et al*. [49]. Briefly, AGS cells were grown overnight in 10 cm culture dish plates and later infected with *H. pylori* cells with a multiplicity of infection of 100∶1 for four hours. After incubation, AGS cells were washed, harvested and fractionated. Membrane fractions were analyzed by Western blotting, using anti-CagA (1∶2000, kindly provided by Antonello Covacci), and anti-phosphotyrosine (1∶2000, anti-PY99, Santa Cruz Biotechnology) antibodies. Analysis with an anti-h-Met antibody (1∶1000, C-28, Santa Cruz Biotechnology) showing the general host membrane protein h-Met served as loading control.

## Supporting Information

Figure S1 WecA alignments. Alignments of WecA polypeptide sequences were done using MultiAlin (http://bioinfo.genotoul.fr/multalin/multalin.html). (A) Alignments of WecA sequences from sequenced *H. pylori* strains G27, 26695, J99, HPAG1 and P12. (B) Alignment of WecA sequences from *H. pylori* G27 and J99 with WecA sequences from *Burkholderia cenocepacia* and *E. coli*.
(0.51 MB GIF)

Figure S2 WaaL alignments. Alignments of WaaL polypeptide sequences were done using MultiAlin (http://bioinfo.genotoul.fr/multalin/multalin.html). (A) Alignments of WaaL sequences from sequenced *H. pylori* strains G27, 26695, HPAG1, P12 and J99. (B) Alignment of WaaL sequences from *H. pylori* G27 and J99 with WaaL sequences from *E. coli*, *Pseudomonas aeruginosa* and *Salmonella typhimurium*.
(0.51 MB GIF)

Figure S3 Lewis antigens are present on the *H. pylori* flagella. Fluorescence microscopy of *H. pylori* J99 using an anti-Le^x^ antibody confirmed the presence of Lewis antigens on the membranous sheath covering the *H. pylori* flagella, indicated by an arrow.
(0.28 MB GIF)

Figure S4 Partial *waaL_HP_* complementation. Purified LPS from 1: wild type *H. pylori* G27, 2: *waaL* mutant, 3: *waaL* complemented was analyzed by Western blot using an anti-Le^y^ antibody. The partial production of smooth LPS in the complemented strain is indicated with an arrow. Protein marker standards were included for reference.
(0.34 MB GIF)

Figure S5 Purification of WaaL_HP_-His_10_. WaaL_HP_-His_10_ purification fractions using nickel affinity chromatography were run on a 10% SDS-PAGE and proteins were stained with coomassie. 1: loading sample; 2: flow through; 3: washing fraction; 4–9: elution fractions 1–6 (1 ml was collected per elution fraction). The band containing WaaL_HP_-His_10_ is indicated with an arrow. Its identity was confirmed by mass spectrometry. Protein marker standards were included for reference.
(0.50 MB GIF)

Figure S6 Validation of the mild acid hydrolysis protocol. (1) *E. coli* LPS, (2) *H. pylori* G27 wild type LPS and (3) *C. jejuni* LLO in the same conditions as applied for the *in vitro* ligation assay are shown in a Western blot using anti-*E. coli* O16 antigen, anti-Le^y^ and HR6 anti-*C. jejuni* glycan antibodies, (A) not hydrolyzed and (B) after mild acid hydrolysis. Mild acid hydrolysis affects UndPP-linked oligosaccharides (lane 3) but does not hydrolyze LPS (lanes 1,2). Protein marker standards were included for reference.
(0.51 MB GIF)

Figure S7 ATP is not required for *H. pylori* WaaL *in vitro* activity. Ligation *in vitro* was performed (1) in the absence and (2) in the presence of ATP (2 mM). Reaction samples were separated with SDS-PAGE (15%) and were analyzed with (A) silver staining and (B, C) Western blotting using the HR6 anti-*C. jejuni* glycan antibody, whereby reaction samples were treated with mild acid in (C), hydrolyzing the UndPP-linked glycan (substrate). Protein marker standards were included for reference.
(0.51 MB GIF)

Figure S8 Wzk alignments. Alignments of translocase polypeptide sequences were done using MultiAlin (http://bioinfo.genotoul.fr/multalin/multalin.html). (A) Alignments of Wzk sequences from sequenced *H. pylori* strains G27, 26695, J99, HPAG1 and P12. (B) Alignment of Wzk sequences from *H. pylori* G27 and J99 with homologous sequences from *Wolinella succinogenes* and *Arcobacter butzleri* and PglK from *C. jejuni*. (C) Alignment of Wzk sequences from *H. pylori* G27 and J99 with homologous sequences from *Wolinella succinogenes* and *Arcobacter butzleri*, PglK from *C. jejuni* and MsbA sequences from *H. pylori* J99 and *E. coli*.
(0.49 MB GIF)

Figure S9 Glycan structures translocated by Wzk. Shown are the glycan structures translocated by Wzk in this study. (A) *H. pylori* O chains containing Lewis antigens. The positions of the fucose residues can change (Skoglund *et al.*, 2009, PLoS ONE). Shown are terminal Le^y^, terminal Le^x^ and internal Le^x^ with terminal Le^y^. (B) *C. jejuni* heptasaccharide (Young *et al.*, 2002, J Biol Chem.). (C) *E. coli* O16 antigen (Stevenson *et al.*, 1994, J Bacteriol.).
(0.17 MB GIF)

Table S1 Oligonucleotides.
(0.04 MB DOC)
