# Supplementary figures and images for: Helicobacter pylori Lipopolysaccharide Is Synthesized via a Novel Pathway with an Evolutionary Connection to Protein N-Glycosylation
Source: PLoS Pathog. 2010 Mar 19;6(3):e1000819. doi: 10.1371/journal.ppat.1000819 (PMC2841628; doi:10.1371/journal.ppat.1000819)

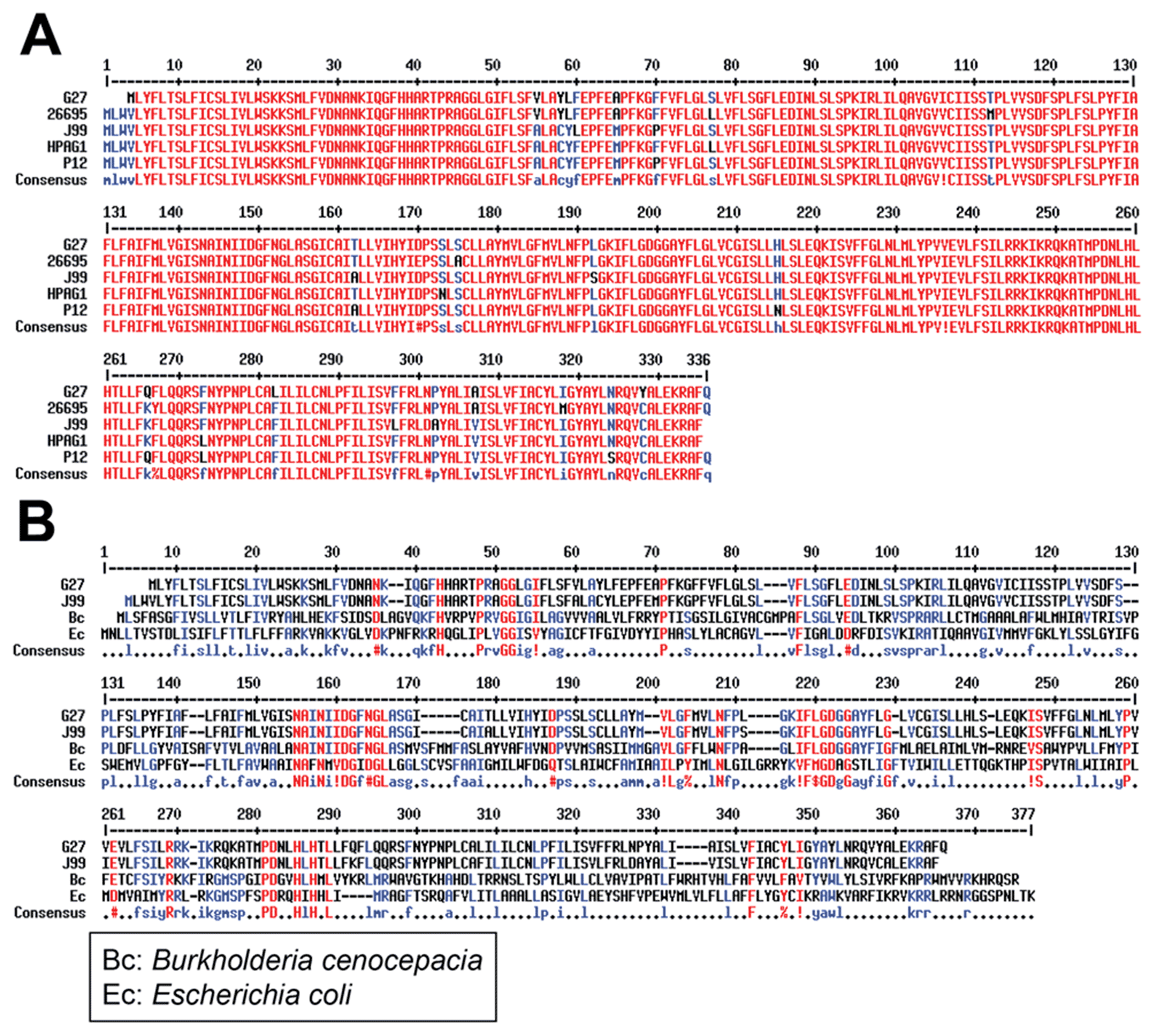

Supplement: Figure S1 — WecA alignments. Alignments of WecA polypeptide sequences were done using MultiAlin (http://bioinfo.genotoul.fr/multalin/multalin.html). (A) Alignments of WecA sequences from sequenced H. pylori strains G27, 26695, J99, HPAG1 and P12. (B) Alignment of WecA sequences from H. pylori G27 and J99 with WecA sequences from Burkholderia cenocepacia and E. coli. (0.51 MB GIF) [file ppat.1000819.s001.gif]

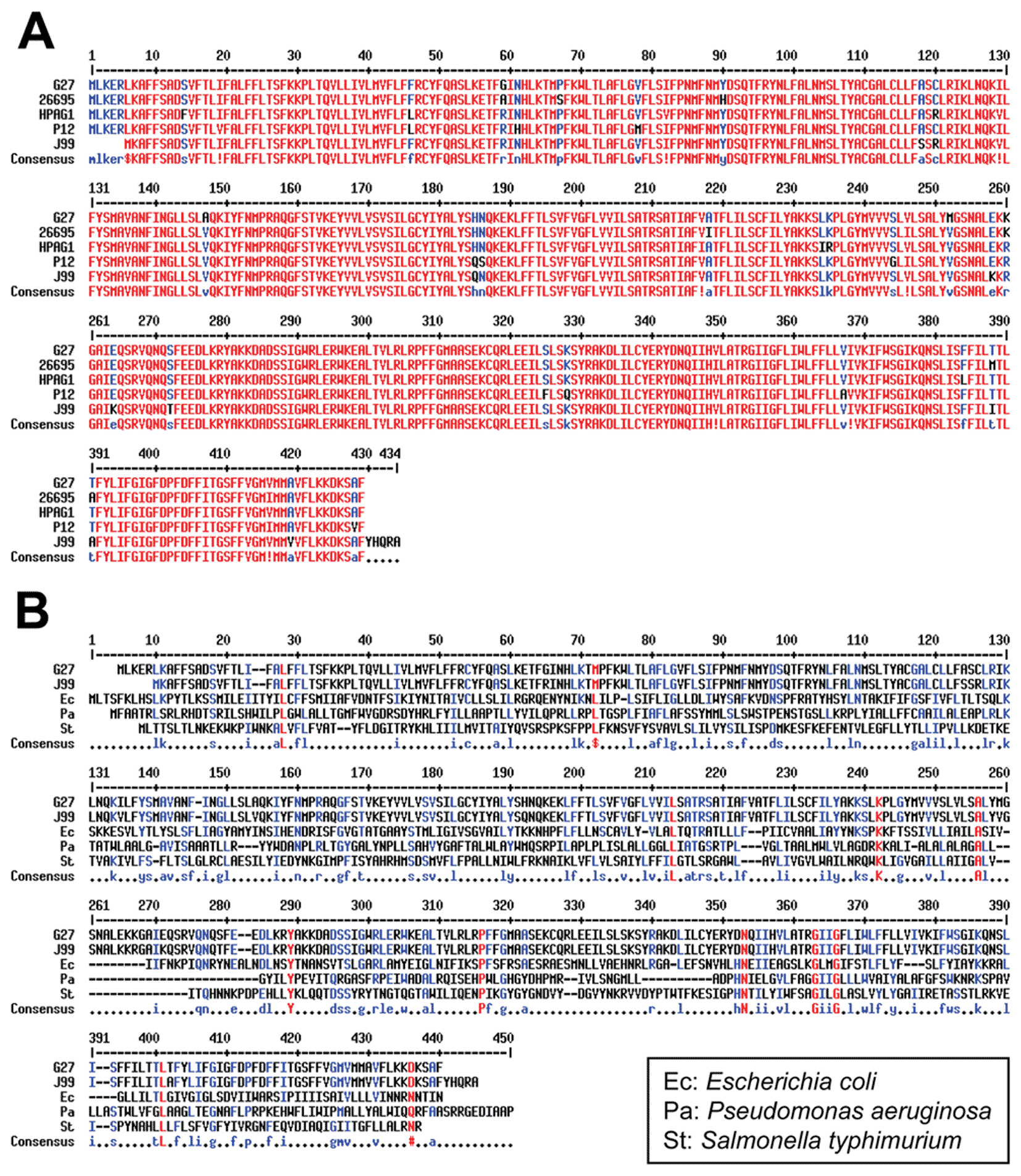

Supplement: Figure S2 — WaaL alignments. Alignments of WaaL polypeptide sequences were done using MultiAlin (http://bioinfo.genotoul.fr/multalin/multalin.html). (A) Alignments of WaaL sequences from sequenced H. pylori strains G27, 26695, HPAG1, P12 and J99. (B) Alignment of WaaL sequences from H. pylori G27 and J99 with WaaL sequences from E. coli, Pseudomonas aeruginosa and Salmonella typhimurium. (0.51 MB GIF) [file ppat.1000819.s002.gif]

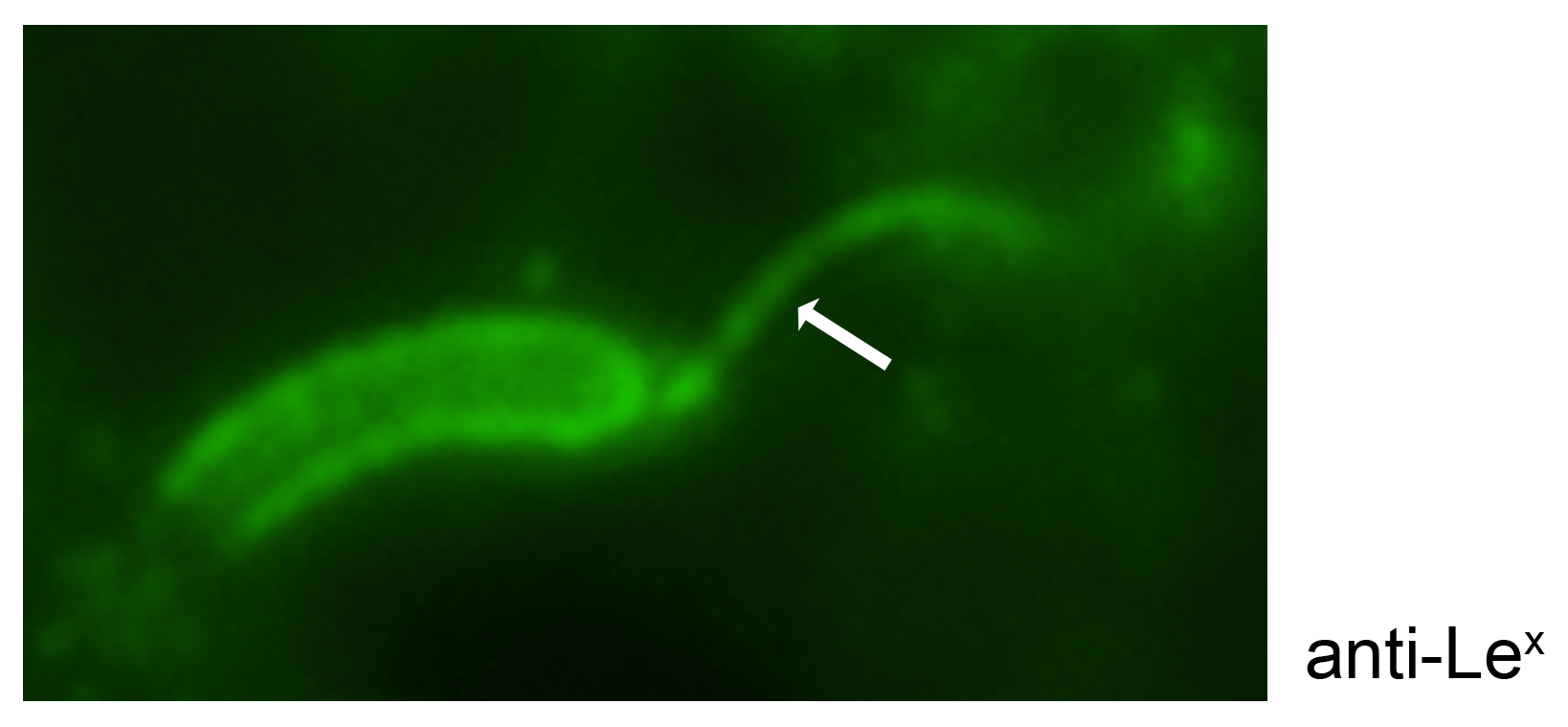

Supplement: Figure S3 — Lewis antigens are present on the H. pylori flagella. Fluorescence microscopy of H. pylori J99 using an anti-Lex antibody confirmed the presence of Lewis antigens on the membranous sheath covering the H. pylori flagella, indicated by an arrow. (0.28 MB GIF) [file ppat.1000819.s003.gif]

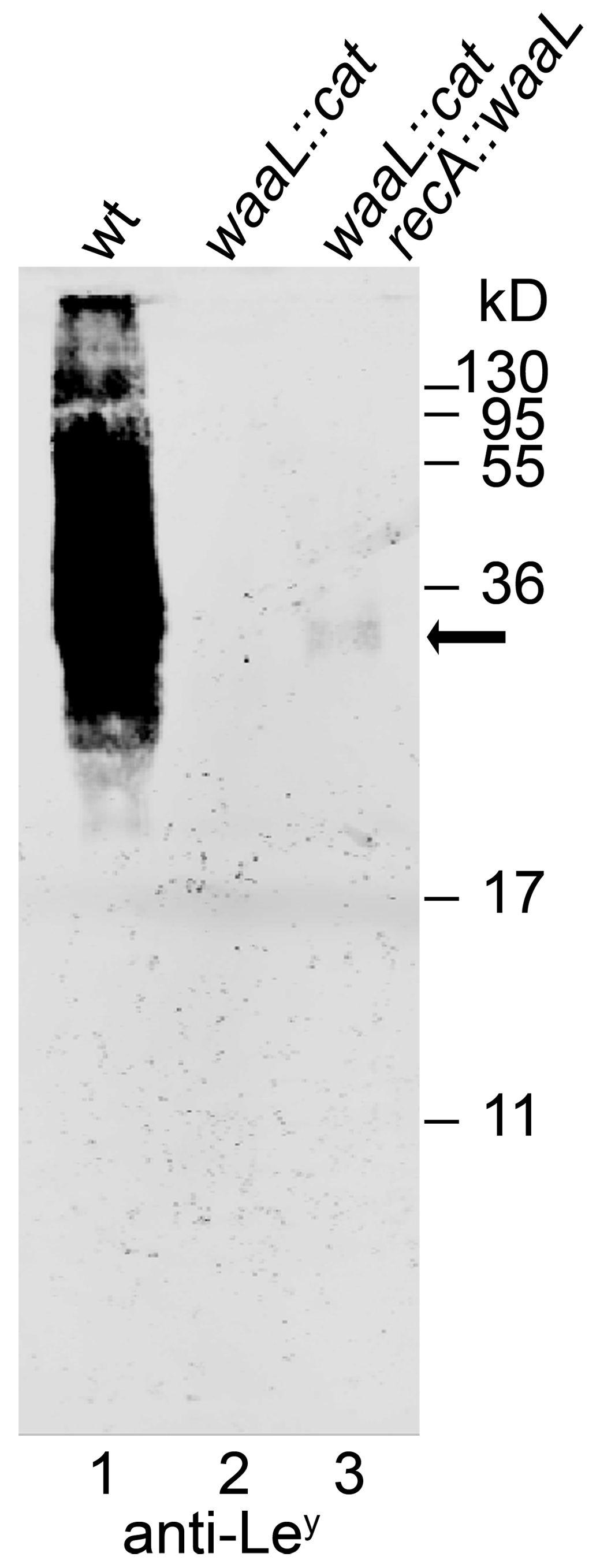

Supplement: Figure S4 — Partial waaLHP complementation. Purified LPS from 1: wild type H. pylori G27, 2: waaL mutant, 3: waaL complemented was analyzed by Western blot using an anti-Ley antibody. The partial production of smooth LPS in the complemented strain is indicated with an arrow. Protein marker standards were included for reference. (0.34 MB GIF) [file ppat.1000819.s004.gif]

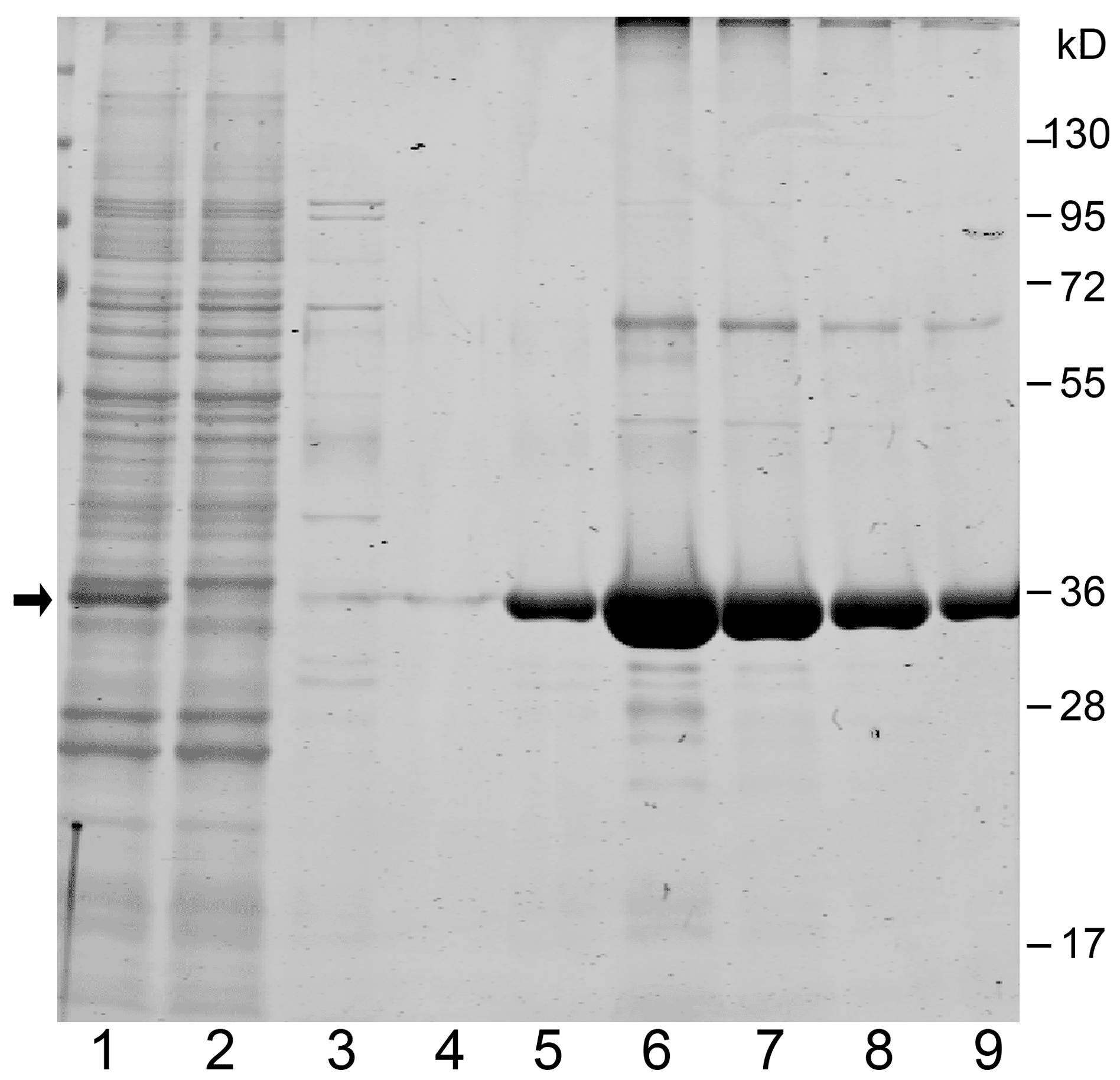

Supplement: Figure S5 — Purification of WaaLHP-His10. WaaLHP-His10 purification fractions using nickel affinity chromatography were run on a 10% SDS-PAGE and proteins were stained with coomassie. 1: loading sample; 2: flow through; 3: washing fraction; 4–9: elution fractions 1–6 (1 ml was collected per elution fraction). The band containing WaaLHP-His10 is indicated with an arrow. Its identity was confirmed by mass spectrometry. Protein marker standards were included for reference. (0.50 MB GIF) [file ppat.1000819.s005.gif]

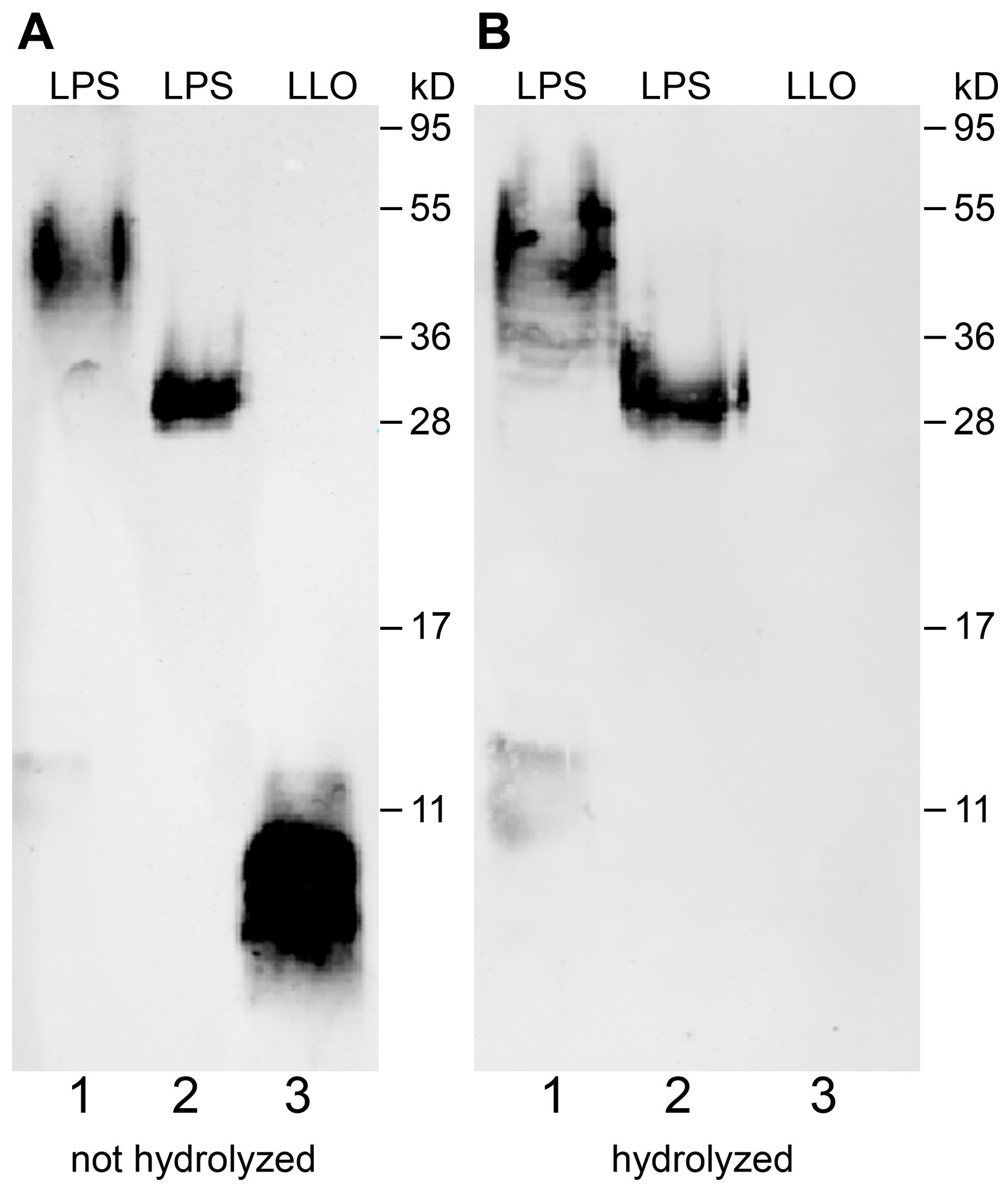

Supplement: Figure S6 — Validation of the mild acid hydrolysis protocol. (1) E. coli LPS, (2) H. pylori G27 wild type LPS and (3) C. jejuni LLO in the same conditions as applied for the in vitro ligation assay are shown in a Western blot using anti-E. coli O16 antigen, anti-Ley and HR6 anti-C. jejuni glycan antibodies, (A) not hydrolyzed and (B) after mild acid hydrolysis. Mild acid hydrolysis affects UndPP-linked oligosaccharides (lane 3) but does not hydrolyze LPS (lanes 1,2). Protein marker standards were included for reference. (0.51 MB GIF) [file ppat.1000819.s006.gif]

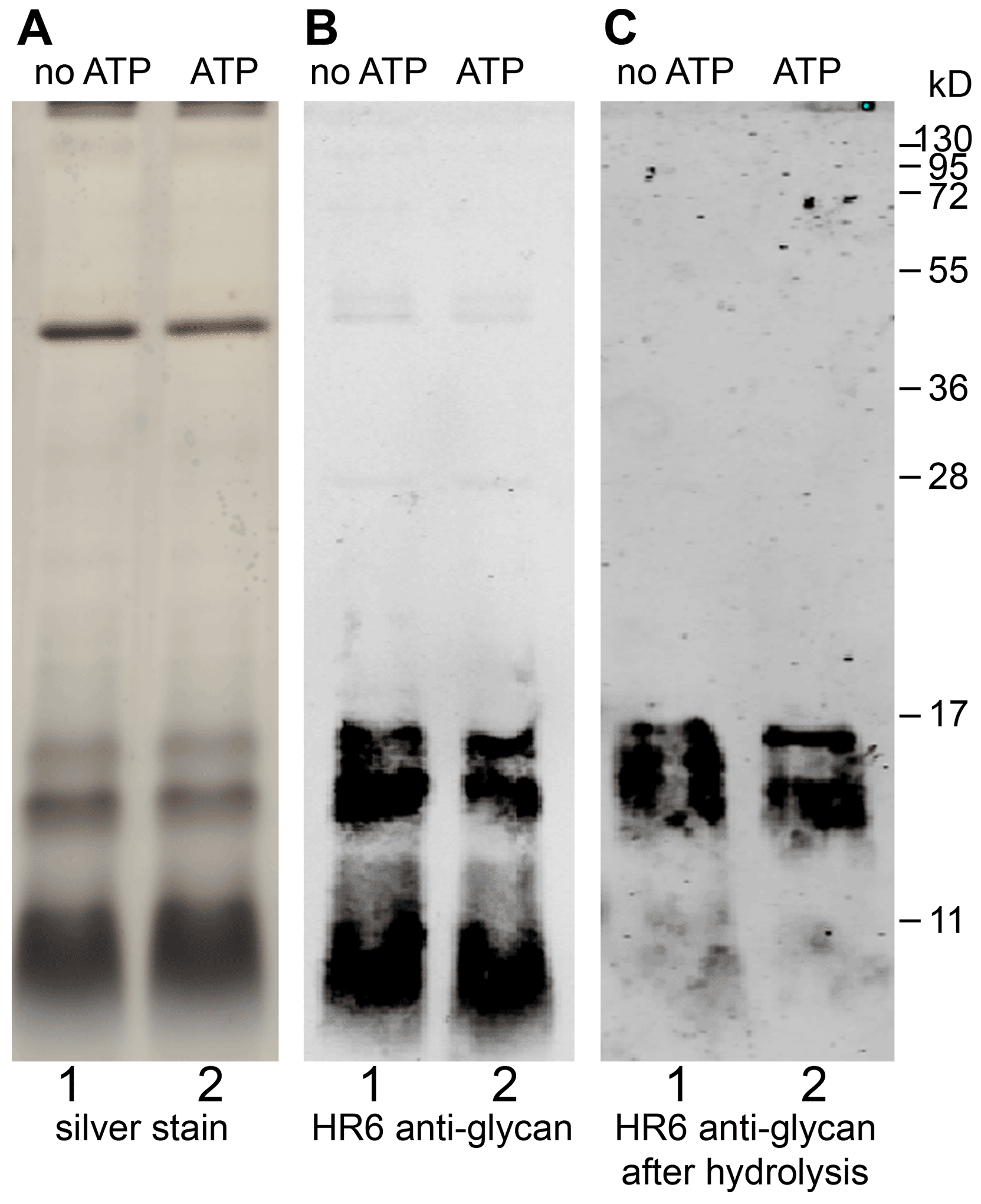

Supplement: Figure S7 — ATP is not required for H. pylori WaaL in vitro activity. Ligation in vitro was performed (1) in the absence and (2) in the presence of ATP (2 mM). Reaction samples were separated with SDS-PAGE (15%) and were analyzed with (A) silver staining and (B, C) Western blotting using the HR6 anti-C. jejuni glycan antibody, whereby reaction samples were treated with mild acid in (C), hydrolyzing the UndPP-linked glycan (substrate). Protein marker standards were included for reference. (0.51 MB GIF) [file ppat.1000819.s007.gif]

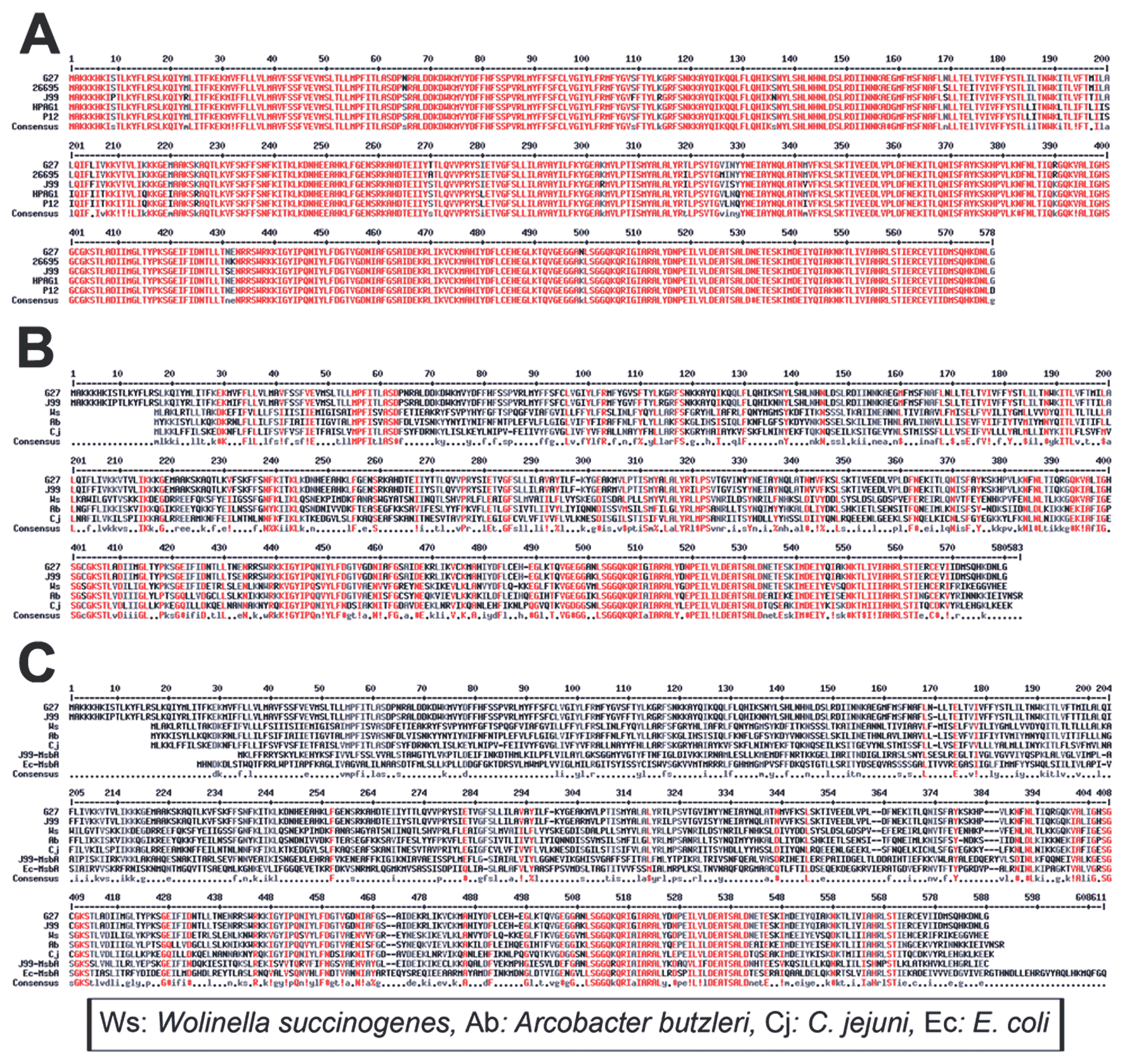

Supplement: Figure S8 — Wzk alignments. Alignments of translocase polypeptide sequences were done using MultiAlin (http://bioinfo.genotoul.fr/multalin/multalin.html). (A) Alignments of Wzk sequences from sequenced H. pylori strains G27, 26695, J99, HPAG1 and P12. (B) Alignment of Wzk sequences from H. pylori G27 and J99 with homologous sequences from Wolinella succinogenes and Arcobacter butzleri and PglK from C. jejuni. (C) Alignment of Wzk sequences from H. pylori G27 and J99 with homologous sequences from Wolinella succinogenes and Arcobacter butzleri, PglK from C. jejuni and MsbA sequences from H. pylori J99 and E. coli. (0.49 MB GIF) [file ppat.1000819.s008.gif]

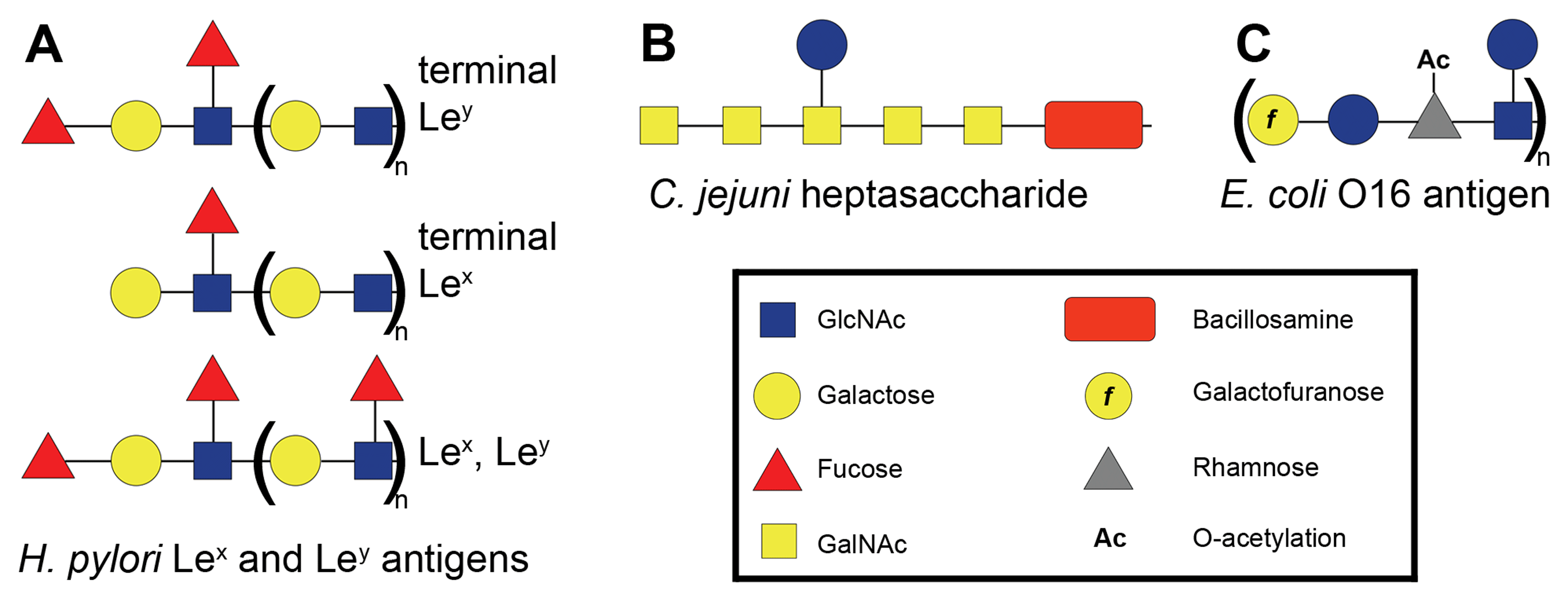

Supplement: Figure S9 — Glycan structures translocated by Wzk. Shown are the glycan structures translocated by Wzk in this study. (A) H. pylori O chains containing Lewis antigens. The positions of the fucose residues can change (Skoglund et al., 2009, PLoS ONE). Shown are terminal Ley, terminal Lex and internal Lex with terminal Ley. (B) C. jejuni heptasaccharide (Young et al., 2002, J Biol Chem.). (C) E. coli O16 antigen (Stevenson et al., 1994, J Bacteriol.). (0.17 MB GIF) [file ppat.1000819.s009.gif]
