# Supplementary material for: Helicobacter pylori Lipopolysaccharide Is Synthesized via a Novel Pathway with an Evolutionary Connection to Protein N-Glycosylation
Source: PLoS Pathog. 2010 Mar 19;6(3):e1000819. doi: 10.1371/journal.ppat.1000819 (PMC2841628; doi:10.1371/journal.ppat.1000819)
Supplement: Table S1 — Oligonucleotides. (0.04 MB DOC) [file ppat.1000819.s010.doc]

Table S1. Oligonucleotides.

| Name | Target | 5’ – 3’ Sequence |
| --- | --- | --- |
| WecAHPEcoRIfw | *H. pylori* J99 *wecA* (JHP1488) | AGAATTCATGTTGTGGGTGCTATATTTTTTAACC |
| WecAHPH6XbaIrv | *H. pylori* J99 *wecA* (JHP1488) | ATCTAGATTAGTGGTGGTGGTGGTGGTGAAACGCCCGCTTTTCTAAAGC |
| WecA_forward | *H. pylori* J99 *wecA* (JHP1488) | GTGTTGTGGGTGCTATATTTTTTAAC |
| WecA_reverse | *H. pylori* J99 *wecA* (JHP1488) | TTAAAACGCCCGCTTTTCTA |
| NdeIHPJ99wecAfw | *H. pylori* J99 *wecA* (JHP1488) | AAACATATGATGTTGTGGGTGCTATATTTTTTAACC |
| HPJ99wecAH6BamHIrev | *H. pylori* J99 *wecA* (JHP1488) | TTTGGATCCTTAGTGGTGGTGGTGGTGGTGAAACG |
| Ligase_forward | *H. pylori* J99 *waaL* (JHP0385) | TTGAAAGCCTTTTTCAGTGC |
| Ligase_reverse | *H. pylori* J99 *waaL* (JHP0385) | TTAAGCCCTTTGATGATAAAAAGC |
| NdeIG27waaLfw | *H. pylori* G27 *waaL* (HPG27_389) | GAGTTTCATATGTTGAAAGAGCGTTTGAAAGC |
| G27waaLH10BamHIrv | *H. pylori* G27 *waaL* (HPG27_389) | AGAAGGATCCCTAGTGATGATGGTGGTGATGGTGGTGATGGTGAAAAGCGCTTTTATCCTTTTTTAAAAAAACAGC |
| KpnIHPpglKfw | *H. pylori* J99 *wzk* (JHP1129) | AAAAGGTACCATGGCGAAAAAAAAACATAAAATTCC |
| HPpglKH6XbaIrev | *H. pylori* J99 *wzk* (JHP1129) | TTTTTCTAGATCAGTGGTGGTGGTGGTGGTGGCCGAGATTGTCTTTGTGTTGG |
| NdeICj81116pglKfw | *C. jejuni* 81116 *pglK* (*wlaB*) | AGAATCACATATGCTAAAAAAACTTTTTTTTATTTTAAGC |
| Cj81116pglKXbaIrv | *C. jejuni* 81116 *pglK* (*wlaB*) | AGAATCTAGATTTCTCCTCTTTAAGCTTACCGTG |
